# Early Diagnosis and Monitoring of Adaptive Immune Response in a Cohort of Mild Mpox Patients During the 2022 Wave

**DOI:** 10.3390/microorganisms13020355

**Published:** 2025-02-06

**Authors:** Sara Caldrer, Silvia Accordini, Annalisa Donini, Natasha Gianesini, Andrea Matucci, Antonio Mori, Cristina Mazzi, Maddalena Cordioli, Evelina Tacconelli, Niccolò Ronzoni, Andrea Angheben, Chiara Piubelli, Federico Gobbi, Concetta Castilletti

**Affiliations:** 1 IRCCS Sacro Cuore Don Calabria Hospital, Via Don A. Sempreboni, 5, Negrar di Valpolicella, 37024 Verona, Italy; 2 Centre for Clinical Research, IRCCS Sacro Cuore Don Calabria Hospital, Negrar di Valpolicella, 37024 Verona, Italy; 3 Division of Infectious Diseases, Department of Diagnostic and Public Health, University of Verona, 37134 Verona, Italy; 4 Division of Infectious Diseases, Department of Medicine, Verona University Hospital, 37134 Verona, Italy

**Keywords:** mpox, non-endemic countries, IgA levels, antibody kinetics, cellular immunity, HIV, Treg

## Abstract

Our study wanted to describe the kinetics of serological and adaptive immune responses in mpox patients. Methods: Fourteen patients with laboratory-confirmed mpox were tested at different time points after the symptom onset. An immunofluorescence assay was performed to evaluate the seroconversion kinetics of specific IgA, IgM, and IgG. Moreover, the characterization of the adaptive immunological profile of T- and B-cells was performed. Results: The antibody kinetics revealed the faster and more effective seroconversion of specific IgA than IgM. Moreover, we detected an increase in Active memory B cells and CD8+ cells in the early phases of infection, and a reduction in CD4+ T-cells in the mpox patients with respect to the controls and found the presence of higher levels of Treg cells in the HIV+ patients in the early phase of infection. Conclusion: Our data highlight the relevance of specific IgA testing early after the symptom onset, suggesting a possible role as a marker in early diagnosis, especially in close contact subjects. Furthermore, the different maturation states of effector cells in HIV+ patients, together with high Treg levels, may lead us to better understand the role of MPXV-HIV co-infection and identify potential cellular markers to monitor the excessive immune activation involved in mpox disease progression.

## 1. Introduction

Mpox is a zoonotic viral disease endemic to Western and Central Africa regions caused by the monkeypox virus (MPXV), which is capable of infecting several animal species. In May 2022, an epidemic of MPXV clade IIb (formerly Western Africa subclade b) also spread in many non-endemic countries outside the African continent through close human–human contact, and on 23 July 2022, mpox was declared a public health emergency of international concern (PHEIC) until 11 May 2023 [1]. This multi-country outbreak recorded 95,226 laboratory-confirmed cases of mpox, including 185 deaths, of which 92,306 were in regions with no history of reported mpox infections [2,3,4]. The end of this emergency was the consequence of the decrease in reported cases; however, MPXV is still continuing to circulate across the world and the current reported global data most likely underestimate the actual number of mpox cases. The World Health Organization (WHO) recommended strict monitoring, together with the possibility of administering the pre- or post-exposure Modified Vaccinia Ankara—Bavarian Nordic (MVA-BN)-based vaccine [5,6].

MPXV infection transmission relies on close contact with infectious material from cutaneous and/or mucosal lesions. Bodily fluids were the key pathway in the widespread human-to-human transmission during this outbreak, and sexual activity has been reported as the main risk factor. Most affected cases during the outbreak were men who have sex with men (MSM) [7,8]. Symptoms are similar but less severe with respect to that reported for smallpox infections [9,10]. MPXV clade IIb infection is rarely fatal and self-resolving. More than 99% of people without severely weakened immune systems survived the infection [11]; however, in this outbreak, HIV+ people have been reported to be disproportionately affected, accounting for 38–50% of mpox patients [12]. These subjects include people living with HIV/AIDS (PLWHA) or using HIV pre-exposure prophylaxis (HIV-PrEP) [13,14]. Most of the reported cases of HIV+ people with high CD4+ cell counts had similar outcomes to those of HIV− subjects.

In contrast, people with an advanced uncontrolled HIV infection had a longer and more severe mpox clinical presentation and also increased mortality than HIV- people [15]. Literature data claim that a previous smallpox vaccination can give about 85% protection against MPXV infection [16]. Unfortunately, the younger population born after 1980 was not immunized due to the discontinuation of vaccination programs after the global smallpox eradication [17], and this may have contributed to the expanded circulation of MPXV. In addition, subclinical or asymptomatic mpox cases could lead to the virus spreading [18], as well as the MPXV’s ability to employ different immune evasion strategies by subverting virus-specific T lymphocyte activity [19].

In this respect, the study of anti-Orthopoxvirus (OPXV) immunoglobulin class kinetics in the acute phase could give some useful insights to support early diagnosis [20,21]. Regarding adaptive cell-mediated immunity, Agrati et al. [22] provided insights into the T-cell-mediated immune response to MPXV infection, demonstrating an early increase in the frequency of CD8+ T-cells with an active phenotype (CD38+) and a decrease in CD4+ T-cell numbers with a normalization 12–20 days after the symptom onset. Also, an increase in inflammatory cytokine concentrations was reported.

Our study wanted to describe the kinetics of serological and adaptive immune responses in patients with MPXV infection in order to implement the current knowledge on the adaptive immune response to the monkeypox virus and its dynamics over time and describe the different maturation states of effector cells in control HIV+ patients during MPXV infection. Understanding humoral and adaptive immunity against MPXV infection could be useful for the interpretation of the disease pathogenesis, vaccine development, and epidemic control measures.

In this study, a cohort of 14 male subjects who tested positive for MPXV infection during the 2022–2023 wave was longitudinally followed after the symptom onset (SO) to better understand the mpox adaptive immune response remodeling. Specific antibody responses and different T- and B-cell subpopulations, as well as activation/exhaustion markers, cell-type specific biomarkers, and cytokine secretion, were analyzed in relation to the patients’ characteristics and disease course. The adaptive immune cell phenotype during the MPXV infection was also analyzed in previously smallpox-vaccinated and HIV+ control patients.

## 2. Materials and Methods

### 2.1. Study Population and Sample Collection

Fourteen adult patients with laboratory-confirmed mpox diagnoses between May and October 2022 were included in this study. The patients were referred to the Department of Diagnostics and Public Health, University Hospital of Verona, or to the Department of Tropical Diseases and Microbiology IRCCS Sacro Cuore Don Calabria Hospital. Demographic and clinical data are reported (Table 1). Four subjects reported to be receiving Anti-Retroviral Therapy (ART) that consisted of a combination of antiretroviral drugs to maximally suppress the virus and to stop the progression of HIV disease, and three subjects reported to have a history of smallpox vaccination (smVAC). In addition, fourteen gender–age-matching healthy donors (HDs) were included in this study as controls. Blood sampling was repeated at different time points after the SO: 10 days ± 3 (T0), 15 days ± 3 (T1), 20 days ± 3 (T2), and 60 days ± 3 (T3). One patient was asymptomatic; in this case, the date of the sample collection corresponded to the date of diagnosis, as they were identified as a close contact of an mpox patient.

### 2.2. Ethics

This study was conducted according to the guidelines of the Declaration of Helsinki. All patients signed a written informed consent form, and the present study received ethical approval from the Ethical Committee of Verona under protocol no. 6232 on 30 January 2023. All the biological samples used for experimental analysis were collected at the time of admission and stored at −80 °C in Tropica Biobank at the IRCCS Sacro Cuore Don Calabria Hospital until use.

### 2.3. Indirect Immunofluorescence Assay

OPXV-specific IgA, IgM, and IgG titers in the serum samples were determined by indirect immunofluorescent assay (IFA) on home-made slides prepared with Vero-E6 cells (ATCC CRL-1586) infected with cowpox virus (CPXV, Taunton strain), as described elsewhere [20,23]. To detect the specific IgA and IgM, the serum samples were pre-treated with an IgG immunosorbent (Eurosorb, Euroimmun, Lubeck, Germany). Each serum was 2-fold serially diluted, starting from 1:20 (screening dilution) and titrated up to 1:1280. In each experiment, anti-human IgA, IgM, and IgG positive control sera and a negative control were included.

### 2.4. Flow Cytometry Analyses on Whole-Blood and Serum Samples

Flow cytometry analyses were performed on the serum and whole-blood samples collected in EDTA aliquoted and stored at −80 °C in 10% DMSO (*v*/*v*) within 6 h from the blood withdrawal. The immunophenotyping was performed on 13 out of 14 patients at three different time points (T0, T1, and T2) after the SO. For the T- and B-cell characterizations, we used the Beckman Coulter DuraClone IM T-cell panel (Beckman Coulter, Miami, FL, USA; Catalog n. B53328) and DuraClone IM B Cells panel (Beckman Coulter, Catalog n. B53318) respectively, as described previously [24].

We also targeted CD4+ sub-populations, including Th1, Th2, Th17, and regulatory T-cells (Tregs), and measured the serum concentrations of twelve human cytokines using the MACSPlex Cytokine 12 kit (Miltenyi Biotec, Bergisch Gladbach, Germany), as recommended by the manufacturer and as previously described [25]. Sample acquisition was performed using a CytoFlex flow cytometer (Beckman Coulter). FCS files were uploaded and analyzed with the Cytobank Premium software (Beckman Coulter) with the CytExpert software v2.3 (Beckman Coulter). The gating strategies are represented in Supplementary Figures S1–S3.

### 2.5. Statistical Analysis

Statistical analyses were performed using Rsoftware v4.2.1 (RCore Team, Vienna, Austria), and the bar/plot graphs were generated with GraphPad Prism v8.3.0 (GraphPad Software, San Diego, CA, USA). Continuous variables were expressed as median and interquartile ranges. Non-parametric tests were applied according to the data distribution. Differences in cell levels were assessed using the Wilcoxon rank-sum test when comparing two groups. The false discovery rate (FDR) correction was used for multiple comparisons. The Spearman coefficient was used to evaluate correlations. The significance level was set at *p*-value < 0.05, and all the tests were two-tailed.

## 3. Results

### 3.1. Population Characteristics

The demographics and clinical characteristics of the mpox patients are reported (Table 1). All patients were male, with a median age of 40.5 years (32.5–46), and 13/14 self-reported as being MSM. All subjects had a PCR-confirmed mpox diagnosis at T0 and presented with a skin rash or lesion, except for one asymptomatic patient. In three cases, the patients declared they were vaccinated with the Variola Vaccine (first generation smallpox vaccine) before the 1980s [26]. Four subjects (28.5%) tested positive for HIV (PLWHA). All the HIV- subjects reported that they took the pre-exposure prophylaxis (or PrEP) for the prevention of HIV infection.

### 3.2. IgA Displayed Early Kinetics During the MPXV Infection

The levels of specific anti-OPXV IgA, IgM, and IgG were tested on serum samples collected between T0 and T3.

**Figure 1:**
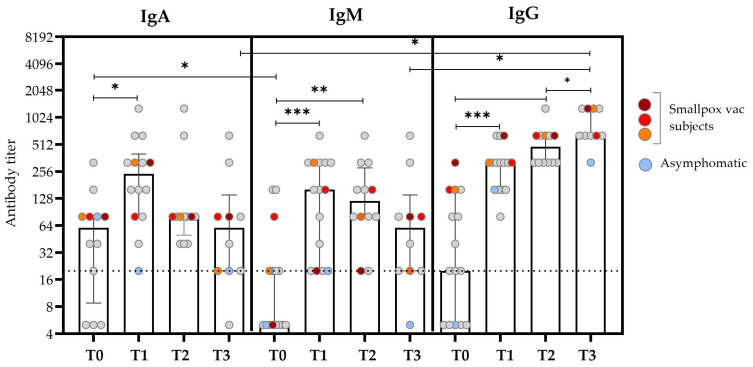
Anti-OPXV IgA, IgM, and IgG antibody titers at different time points after the SO. The bars represent the medians and IQRs for all the patients, while the scattered plots show individual measurements. The red, orange, and dark red plots represent the antibody titers of patients who reported a history of smallpox vaccination. The light blue plots represent values obtained from the asymptomatic subjects. The dashed line represents the threshold for the limit of detection (based on the screening dilution) for the antibody titer. Non-smVAC HD subjects presented an antibody titer ≤ 1:20. Comparisons were made using the Wilcoxon signed-rank test for paired data with a false discovery rate correction (fdr). Significant differences were represented as follows: * *p* ≤ 0.05, ** *p* ≤ 0.01, and *** *p* ≤ 0.005.

The kinetics of the IgA titer was similar to that observed for IgM, with the median value increasing early after the SO (T0 vs. T1, *p* = 0.020), and it reached the peak after about 15 days after the SO (T1) and then started to decrease after about 20 days (T2) (Figure 1 and Table 2). Notably, the IgA antibody titer at T0 was significantly higher than that of IgM (*p* = 0.041); this difference disappeared over time. IgM was typically absent or present at low levels in the first days after the SO; the titer began to rise during the second week after the SO and then decreased to basal levels at T3.

The kinetics of the IgG titers presented the lower titer at T0, followed by a continuous increase over time, which reached the maximum value at T3 after the SO (*p* = 0.012). In the previously smallpox-vaccinated subjects, an earlier IgG antibody response was observed at T0 compared with the unvaccinated subjects (colored plots in Figure 1). Considering the role of HIV co-infection in the antibody kinetics, no significant differences were found in the antibody titers evaluated at different time points [21].

### 3.3. Kinetics of Activated B-Cells in Mpox Patients

**Figure 2:**
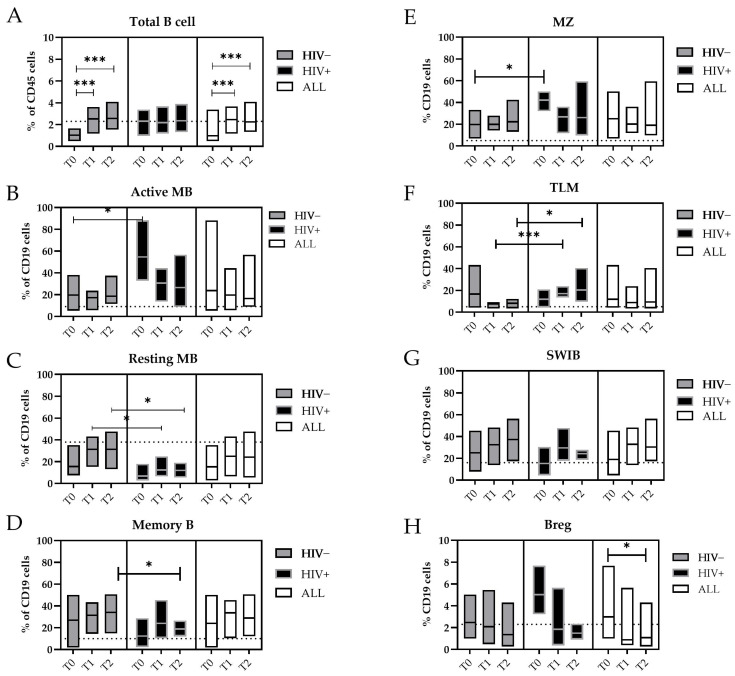
Changes in the B-cell subpopulations during the MPXV infection. Floating bars represent the median and min and max frequency values of each B-cell subpopulation regarding the CD19+ cells. The dashed lines represent the median value obtained from HDs. The white bars represent all mpox subjects included in this study, the gray bars only the HIV− subjects, and the black bars the HIV+ subjects. Statistical differences between the groups were determined using the Wilcoxon rank-sum test. Significant differences were represented as follows: * *p* ≤ 0.05, and *** *p* ≤ 0.005. Cells were indicated as: (**A**) Total B cells (CD19+); (**B**) Active MBs (**B**): Active memory B-cells (IgD−/CD27+/CD21−); (**C**) Resting MBs: Resting Memory B-cells (IgD−/CD21+/CD27+); (**D**) Memory Bs (IgD−/CD27+); (**E**) MZ: marginal zone (CD27+/IgD+); (**F**) TLMs: tissue-like memory B-cells (CD21−/CD27−); (**G**) SWIBs: Switched B-cells (CD27+/IgD−/IgM−); (**H**) B regs: B regulatory B-cells (Cd19+/CD24 high/CD38 high).

The analysis and quantification of maturation stage markers in the circulating B-cells were conducted using the gating strategy illustrated in Figure S1. The frequencies of all the B-cell sub-populations in the mpox subjects with or without HIV co-infection are presented in Figure 2, and numerical values are reported (Tables S1 and S2).

During the MPXV infection, we measured a significant increase in the frequency of the total B-cells from T0 to T2 (T0 vs. T1, *p* = 0.005; T0 vs. T2, *p* = 0.004) (Figure 2A). Interestingly, the circulating Active Memory B-cell (Active MB, IgD−/CD21−/CD27+) values measured during the early phase of infection (T0) were significantly higher in the HIV+ patients (median: 43.0%) in comparison with the HIV- patients (median: 18.4%, *p* = 0.018) or HDs (median: 4.9%) (Figure 2B).

Otherwise, the levels of the Resting Memory B-cells (Resting MBs, IgD−/CD21+/CD27+) were lower, increased with the temporal distance from the SO, and reached the values of the HDs (median: 37%). However, in HIV+ individuals, these levels persisted at significantly lower levels at both T1 and T2 (median: 12.4%) compared with those who were HIV− (T1 and T2 median: 31.4%; *p* = 0.05 and *p* = 0.016, respectively) (Figure 2C). Furthermore, the proportion of memory B-cells (MBs) increased during the MPXV infection, where they reached 35.2% of the B-cells in the HIV+ subjects, whereas in the HIV− subjects, they did not exceed 18.3% of the B-cells at T2 (*p* = 0.048) (Figure 2D).

As for the Active MBs, the marginal zone (MZ) B-cells were higher during the early phase of the MPXV infection in the HIV+ subjects (T0, median: 44.2%) than in the HIV− patients (T0, median: 17.2%; *p* = 0.018) (Figure 2E). The tissue-like memory (TLM) B-cells CD21−/CD27− were more prevalent in the peripheral blood of HIV+ patients compared with the HIV- patients at T1 (median: 15.4% vs. 8.6%, *p* = 0.003) and T2 (median: 15.7% vs. 8.5%, *p* = 0.028) and the HDs (median: 6.2%) (Figure 2F).

The general active involvement of the humoral compartment in the MPXV infection was supported by the expansion of the Switched B-cells (SWIBs, CD27+/IgD−/IgM−) from T0 to T2, which were not affected by the HIV coinfection (Figure 2G). Higher levels of B regulatory cells (B regs, CD24 high/CD38 high) were detected in the early phase of the MPXV infection (T0 = 3.0% vs. T2 = 1.1%, *p* = 0.017) (Figure 2H).

### 3.4. The T-Cells’ Immune Signature During MPXV Infection

**Figure 3:**
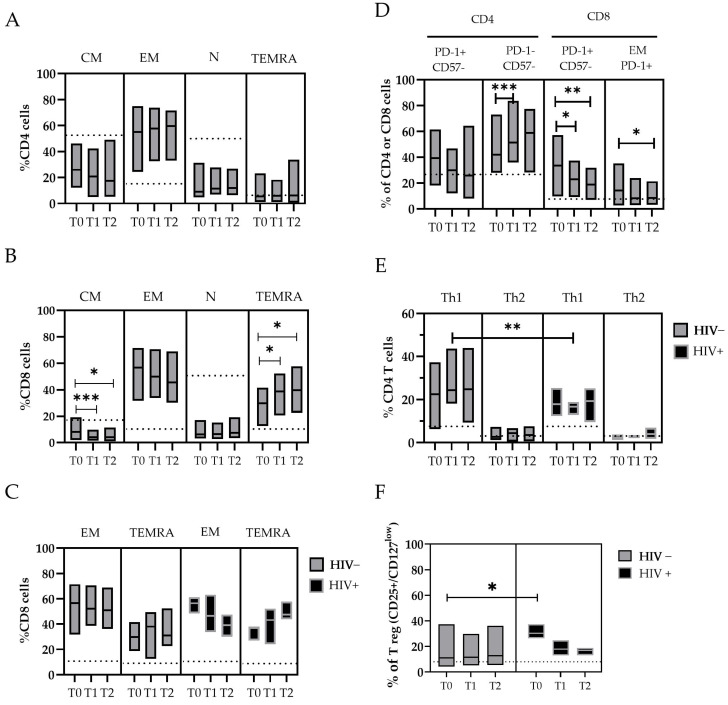
The changes in T-cell subpopulations during the MPXV infection were described in panel (**A**–**F**). The floating bar represents the median and min and max frequency values of each T-cell subpopulation in relation to the time point after the SO and the HIV coinfection. The dashed line represents the median value obtained from the HDs. Statistical differences between the groups were determined using the Wilcoxon rank-sum test. Significant differences are represented as follows: * *p* ≤ 0.05, ** *p* ≤ 0.01, and *** *p* ≤ 0.005. T-cells are indicated as N: Naive (CD45RA+ CCR7+); CM: Central Memory T-cell (CD45RA− CCR7+); EM: Effector Memory T-cell (CD45RA− CCR7−); TEMRA: Effector Memory-expressing CD45RA T-cell (CD45RA+ CCR7−) in both CD4+ and CD8+ populations; Th1: T helper cells (CD4+) type 1; Th2: T helper cells (CD4+) type2; and Treg: T regulatory T-cell (CD25+/CD127 low).

An immunophenotypic analysis of peripheral circulating T-cell populations in MPXV-infected individuals and HDs was performed (Figure 3, Figures S2 and S3, Tables S1 and S2). As previously stated by Agrati et al. [22], patients with a confirmed MPXV infection showed a significantly lower frequency and cell count of CD4+ T-cells and a higher percentage of CD8+ T-cells early after the SO compared with the HDs (as described in Supplementary Figure S4).

Considering the maturation state of T-cells, we observed a lower frequency of Naïve (N-CD4+) and Central memory (CM-CD4+) cells in patients with mpox compared with the HDs, and an increase in Effector memory (EM-CD4+) and TEMRA-CD4+ cells that started from T0 (Figure 3A). Similar observations are reported for the CD8+ cytotoxic T lymphocyte (CTL) subpopulations, where a lower frequency of N-CD8+ cells and a significant decrease in CM-CD8+ cells over time (T0 median: 8.2 vs. T1: 4.2, *p* = 0.007; T0 vs. T2, *p* = 0.02) were found in the mpox patients. Conversely, higher levels of EM- and TEMRA-CD8+ T-cells were measured in mpox patients compared with the HDs, with a significant increase in the TEMRA-CD8+ T-cells over time (T0 median: 29.9% vs. T1: 38.7%, *p* = 0.035; T0: 29.9% vs. T2: 39.6%, *p* = 0.045) (Figure 3B). Interestingly, the subset EM-CD8+ showed a rapid decrease over time in the HIV+ subjects (T0 median: 56.5% vs. T2: 39.2%) compared with the HIV− subjects (T0 median: 57.0% vs. T2: 50.8%). Meanwhile, we observed an increase in the TEMRA-CD8+ cells in the HIV+ subjects (T0 median: 36.9% vs. T2: 47.5%) compared with the HIV− subjects (T0, median: 29.3% vs. T2: 31.0%) (Table S2 and Figure 3C).

An in-depth analysis of the expression of exhaustion and senescence markers (CD57/PD-1) was also performed for all the T-cell subsets. For the CD8+ cells, we observed a higher frequency of PD-1+ in the mpox subjects at T0 (33.5%), with a reduction over time after 20 days (T1 median: 22.9%, *p* = 0.046; T2: 18.7%, *p* = 0.020). These cells were mostly represented by EM-CD8+ PD-1+ cells (T0 median: 15.2% vs. T2: 8.3%, *p* = 0.05) (Figure 3D).

The balance between different reacting T helper populations (Ths) was also investigated. We analyzed the Th CD4+CCR6− cells with phenotypes Th1 (CCR4−/CXCR3+), Th2 (CCR4+/CXCR3−), and Tregs expressing CD25 and CD127 low markers (Figure 3E,F). The amount of Th1 cells was higher in all the mpox patients. In particular, the levels of Th1 cells were higher in the HIV+ subjects than in the HIV− subjects after approximately 10 days after the SO (T1 median: 24.4% vs. 16.7%, respectively; *p* = 0.011) (Figure 3E).

To evaluate the role of a chronic co-infection during the MPXV challenge, we measured the abundance of Tregs (CD25+/CD127 low), both as a number of events and relative percentage of the total CD4+ cells. The Treg cells were significantly upregulated during the early phases of the mpox disease, especially in the HIV+ subjects (T0 = 30.3% of CD4+) compared with the HIV− subjects (T0 = 11.0%, event count difference *p* < 0.01) (Figure 3F), with a trend of normalization with the resolution of infection. High levels of IL-6 were measured in the mpox-infected subjects early after the SO, with a wide variability between the patients [range 6–798 pg/mL] that decreased over time; in contrast, the IFN-α and IFN-γ concentrations were not significantly elevated in our patient cohort (Table S1).

## 4. Discussion

This study focused on the dynamics of serological and adaptive immune responses in a group of patients with mild mpox during the 2022 outbreak [27]. Longitudinal immune profiling aimed to describe the engagement of B- and T-cell responses during this MPXV virus challenge also in the presence of previous vaccination and HIV-controlled infections, and to assess methods for rapid and effective diagnoses. Our study emphasized the important role of IgA in early diagnosis, with the IgA kinetics characterized by a significant early increase in the antibody titer compared with IgM and IgG. Moreover, the HIV infection did not influence the antibody titer levels at different time points, as previously observed [21] Considering these factors, we recommend the measurement of anti-OPXV IgA antibody titers as an informative aid for the early diagnosis of MPXV infection, especially in close contacts, in order to identify individuals with an asymptomatic infection and to help prevent the spread of the disease.

Furthermore, to identify the cellular biomarkers that could provide unique insights into the disease’s progression, the alterations in the adaptive immune cell phenotypes during the MPXV infection were evaluated while also considering the role of previous HIV infection. To date, consistent data of several B-cell abnormalities in HIV infection have been reported [28,29], but scarce information are available about the in vivo kinetics of B-cell responses in HIV+ mpox patients. In our mpox patients’ cohort, we observed a different B-cell profile in the HIV+ subjects with respect to the HIV− subjects. We detected high levels of Active MBs early after the SO and persistent lower levels of Resting Memory B-cells in the HIV+ individuals compared with those in the HIV− individuals up to thirty days after the SO. Moreover, we observed elevated numbers of tissue-like memory (TLM) B-cells in the HIV+ subjects, as was already described for several infectious and non-infectious settings with chronic activation of the immune system and inflammation [30]. Additionally, we measured high levels of B regulatory cells in the early phase of the MPXV infection, particularly in the HIV+ patients compared with the HIV− patients [28]. These data confirm the active involvements of B-cells and humoral compartments in the immune response to MPXV infection and highlight some specific signatures in B-cell phenotypes in mild symptomatic mpox patients bearing chronic HIV infection.

Furthermore, we observed the changes in the T-cells repertoire and demonstrated a rapid and effective T-cells response as a marked reduction of naive CD4+ and CD8+ T-cells and a spreading of the Effector and Terminally Differentiated Effector CD8+ phenotype. We also measured a prevalence of the Th1 phenotype in CD4+ cells and an increased expression of PD-1 in CD8+ cells early after SO, suggesting a highly engaged immune system that showed an expansion phase and culminated in the generation of effector T-cells, which conformed with what has already been reported [22,31].

However, the measurements of circulating cytokines showed low levels of all cytokines, except for IL-4 and IL-6, which were similar to those found in patients with mild symptoms [32]. Literature data indicate that several Orthopox viruses modulate the host immune response: impairing the Natural Killer cells function and reducing the secretions of TNF-α, IFN-α, and IFN-γ (associated with Th1 function), with an opposite increase in Th2-associated cytokines (IL-4, IL-6, IL-5, IL-8) [33]. Moreover, diminished levels of IFN, TNF, and IL-2 display an anti-inflammatory milieu based on regulatory T-cells [33,34].

Therefore, our data describe an immune profile and a cytokine asset that promotes antibody production and T-cell activation that is able to overcome the MPXV immune evasion attempt, which should be consistent with mild human MPXV infections. Despite the people in our cohort who were living with HIV presenting a well-controlled viremia, we described some perturbations in the cellular immunophenotype that were consistent with the HIV chronic infection [35]. For instance, the ratio CD4+/CD8+ was lower in the HIV+ subjects (median 0.6 [0.4–0.6]) early after the SO at T0, with a normalization over time (Table S2) [36].

Moreover, in the HIV+ subjects, we observed a higher frequency of Treg cells in the acute phases of the MPXV infection. As previously described, viruses such as HIV and HCV modulate the host immune system to promote Treg expansion to favor virus persistence through immune subversion [37,38]. Treg cells can play critical roles by limiting excessive immune activation and tissue damage, while at the same time, facilitating pathogen persistence and maintenance of immunity, especially in chronic viral infections [39]. Therefore, Treg cells could be an interesting marker to monitor the immunological state during the mpox acute viral infection in cases of chronic co-infections.

Summarizing, in our cohort of mild symptomatic mpox patients, we observed an effective adaptive immune response that can protect against the onset of a severe clinical picture. This response is efficient, regardless of the presence of immune dysregulation due to HIV infection. However, our results show that MPXV and HIV co-infection displayed some immune signatures indicating that the HIV-1 chronic infection, even if well controlled, may affect the initial response to MPXV infection, but was able to resolve the infection anyway.

The small number of patients analyzed was a limitation of this study. It will be necessary to evaluate a larger cohort of clinical cases of monkeypox in the current outbreak, particularly to verify the observations about HIV co-infection. As a future perspective, we will examine the short-term T-cell response following stimulation with the MVA peptide, taking into account the existing results on vaccination, in order to develop models of long-term immune protection.

## 5. Conclusions

Understanding humoral and adaptive immunity against MPXV is essential for interpreting the disease pathogenesis, developing vaccines, and implementing epidemic control measures. The added value of this study is that it highlights the significance of specific IgA testing in the early stages of the SO, indicating its potential as a marker for early diagnosis, especially in close-contact subjects.

Furthermore, the observed differences in the early immune responses in the HIV control patients during the MPXV infection suggest that the identification of potential cellular markers, such as T-regulatory cells, could be useful for monitoring the excessive immune activation and tissue damage involved in the disease progression.

## Figures and Tables

**Table 1:** Baseline demographic and clinical characteristics of the mpox patients included in this study.

Subject ID	#1	#2	#3	#4	#5	#6	#7	#8	#9	#10	#11	#12	#13	#14	All
Age (years)	31	45	36	43	31	48	39	32	46	44	42	47	23	26	40.5
Gender	M	M	M	M	M	M	M	M	M	M	M	M	M	M	M
MSM	Y	N	Y	Y	Y	Y	Y	Y	Y	Y	Y	Y	Y	Y	13/14
HIV status	+	−	−	−	+	−	−	+	−	−	−	−	−	+	4/14
ART	Y	N	N	N	Y	N	N	Y	N	N	N	N	N	Y	4/14
Smallpox vaccination history (smVAC)	N	Y	N	N	N	Y	N	N	N	N	N	Y	N	N	3/14
Skin lesions/skin rash	Y	Y	Y	N	Y	Y	Y	Y	Y	Y	Y	Y	Y	Y	13/14
Lymphoadenopathy	Y	N	N	N	Y	Y	Y	Y	N	Y	Y	Y	N	N	9/14
Systemic symptoms (fever, myalgia, asthenia, headache)	Y	Y	Y	N	Y	Y	Y	Y	Y	Y	Y	Y	Y	Y	13/14
PCR + (at diagnosis) on															
Lesions	Y	Y	Y	N	Y	Y	Y	Y	Y	Y	Y	Y	Y	Y	13/14
Throat swab	Y	Y	NT	N	Y	Y	Y	N	Y	N	Y	Y	Y	Y	10/14
Anal swab	Y	NT	N	Y	NT	Y	Y	Y	NT	N	N	Y	Y	Y	8/14

NT: not tested; Y: yes; N: no; +: HIV positive; −: HIV negative.

**Table 2:** Serological levels of specific anti-OPXV IgA, IgM, and IgG at different time points after SO in mpox subjects.

Anti-Orthopox Antibodies		Time Points				Multiple Comparison *p*-Value (*)					
		T0 N = 14	T1 N = 14	T2 N = 12	T3 N = 10	T0 vs. T1 N = 14	T0 vs. T2 N = 12	T1 vs. T2 N = 12	T0 vs. T3 N = 10	T1 vs. T3 N = 10	T2 vs. T3 N = 10
IgM Median (IQR)		5 (5–20)	160 (20–320)	120 (80–280)	60 (20–140)	**0.002**	**0.006**	0.611	0.313	0.392	0.130
IgG Median (IQR)		20 (5–100)	320 (160–400)	480 (320–640)	640 (640–1280)	**0.001**	**0.004**	0.063	**0.012**	**0.013**	**0.038**
IgA Median (IQR)		60 (12.5–80)	240 (80–400)	80 (50–80)	60 (20–140)	**0.020**	0.362	0.362	1.00	0.149	0.489
**Multiple comparison** *p*-value (*)	**IgA vs. IgG**	0.385	0.637	0.112	**0.014**						
	**IgA vs. IgM**	**0.041**	0.252	0.838	0.402						
	**IgM vs. IgG**	0.162	0.252	0.056	**0.014**						

Wilcoxon signed-rank test for paired data with false discovery rate correction (fdr). (*) Significant *p*-values are shown in bold. See Figure 1 for details on significant comparisons.

## Data Availability

The de-identified dataset containing demographic information and experimental data are available in the Zenodo repository: https://doi.org/10.5281/zenodo.14288282.

## Supplementary Materials

The following supporting information can be downloaded at: https://www.mdpi.com/article/10.3390/microorganisms13020355/s1, Table S1: B- and T-cell subtype frequencies according to the time points after the SO; Table S2: B- and T-cell subtype frequencies according to the HIV coinfection and the time after the SO; Supplementary Figure S1: Gating strategy for B-cell phenotypes; Figure S2: Gating strategy for CD4 and CD8 T-cell phenotypes. Figure S3: Gating strategy for CD4 Th1/Th2/Th17 and Treg phenotypes. Figure S4: The CD4+ and CD8+ T cells count and frequency during MPXV infection.
