# Supplementary material for: Early Diagnosis and Monitoring of Adaptive Immune Response in a Cohort of Mild Mpox Patients During the 2022 Wave
Source: Microorganisms. 2025 Feb 6;13(2):355. doi: 10.3390/microorganisms13020355 (PMC11858686; doi:10.3390/microorganisms13020355)
Supplement: Supplementary file 1 [file microorganisms-13-00355-s001.zip › Caldrer_Suplementary materials_v.3.pdf]

Supplementary tables to the manuscript entitled: Early diagnosis and monitoring of adaptive immune response in a cohort of mild mpox patients during the 2022 wave

By Sara Caldrer[1*], Silvia Accordini[1], Annalisa Donini[1], Gianesini Natasha[1], Andrea Matucci[1], Antonio Mori[1], Cristina Mazzi[2], Maddalena Cordioli[3,4], Evelina Tacconelli[4], Niccolò Ronzoni[1], Andrea Angheben[1], Chiara Piubelli[1], Gobbi Federico[1], Concetta Castilletti[1*]

Supplementary Tables

Table S1: B and T cell subtype frequencies according to the time points from SO

	Time points			Multiple comparisons' p-value[1]		
**Characteristic**	**T0, N = 12**	**T1, N = 13**	**T2, N = 12**	**T0-T1**	**T0-T2**	**T1-T2**
B event count	1,008.5 (590.2- 1,027.2)	1,028.0 (1,008.0- 1,094.0)	1,033.5 (1,020.2- 1,128.0)	0.068	0.026	0.6
% B cells on Leuko	1.0 (0.8- 1.6)	2.5 (1.7- 3.2)	2.2 (1.6- 3.3)	**0.005**	**0.004**	0.9
Active MB (IgD-/CD27+/CD21-)	23.7 (15.7- 34.2)	19.5 (15.4- 23.6)	16.5 (13.4- 22.4)	0.6	0.4	0.6
B reg	3.0 (1.4- 4.2)	0.9 (0.5- 3.1)	1.1 (0.6- 1.7)	0.077	**0.017**	0.6
CD27-	46.5 (37.2- 55.0)	46.7 (40.1- 59.6)	49.8 (39.7- 56.9)	0.6	0.7	>0.9
CD27+	53.4 (45.3- 62.9)	53.4 (40.4- 60.1)	50.2 (43.2- 60.5)	0.6	0.7	>0.9
DN (IgD-/CD27-)	7.6 (6.7- 9.7)	8.1 (5.9- 8.6)	8.5 (6.3- 8.9)	>0.9	>0.9	0.7
MZ (IgD+/CD27+)	25.1 (14.7- 32.4)	20.2 (17.1- 27.8)	19.1 (15.5- 24.3)	0.6	0.7	0.8
Memory B (IgD-/CD27+)	24.0 (6.6- 35.4)	33.6 (17.0- 40.2)	28.9 (17.1- 37.8)	0.3	0.4	>0.9
Naive (IgD+/CD27-)	43.8 (30.2- 50.0)	40.2 (35.3- 49.6)	38.5 (29.3- 47.1)	>0.9	0.8	0.8
plasmablasts (SWI/CD38+)	0.5 (0.3- 1.5)	0.3 (0.1- 1.1)	0.3 (0.2- 1.0)	0.4	0.3	>0.9
Resting MB (IgD-/CD27+/CD21+)	15.3 (11.3- 21.2)	24.9 (16.0- 32.3)	24.2 (14.8- 33.3)	0.14	0.2	>0.9
SWI (CD27+/ IgD-/IgM-)	19.0 (14.2- 30.6)	32.8 (19.8- 41.1)	30.3 (23.2- 42.6)	0.068	0.068	0.8
TLM (CD21-/CD27-)	11.9 (8.6- 21.0)	8.8 (7.9- 13.5)	9.2 (8.2- 10.9)	0.2	0.3	0.8
Trans B (CD27-/CD24+/CD38+)	1.8 (0.7- 3.9)	0.4 (0.2- 2.5)	0.6 (0.1- 1.3)	0.2	0.040	0.4
UNSW (CD27+/IgD+/IgM+)	68.2 (56.2- 76.5)	58.6 (51.3- 74.8)	62.7 (53.5- 68.4)	0.3	0.4	0.8
CD4 event count	3321.5 (2,943.5- 3,593.5)	3,447.0 (2,824.0- 3,780.0)	3,653.5 (3,263.0- 4,446.2)	0.6	0.13	0.4
% CD4 on CD3	34.5 (31.6- 43.7)	37.4 (34.5- 44.0)	40.1 (35.2- 42.5)	0.5	0.6	>0.9
CD4+ EM1	22.7 (16.2- 29.2)	26.2 (22.0- 33.7)	26.6 (18.7- 39.1)	0.3	0.3	0.9
CD4+ EM2	2.6 (1.0- 8.0)	2.8 (0.8- 5.1)	2.4 (1.2- 10.6)	>0.9	0.9	0.9
CD4+ EM3	12.6 (3.3- 18.6)	11.2 (2.5- 14.4)	7.8 (3.0- 18.5)	>0.9	0.8	0.8
CD4+ EM4	8.6 (5.9- 11.2)	8.9 (6.4- 11.5)	7.7 (6.3- 11.3)	0.6	0.8	0.6
CM-CD4+	26.0 (19.8- 36.8)	20.9 (13.9- 34.9)	17.4 (10.3- 38.5)	0.2	0.2	0.8

EM-CD4+	55.1 (37.9- 63.4)	57.7 (41.4- 69.5)	59.6 (45.7- 66.9)	0.4	0.5	0.8
N-CD4+	9.1 (6.1- 20.6)	11.4 (7.6- 16.7)	11.9 (10.2- 14.6)	0.4	0.4	>0.9
TEMRA-CD4+	5.5 (1.9- 11.8)	6.0 (3.4- 11.8)	6.2 (4.1- 12.3)	0.7	0.7	0.9
CD4+ CD27-/CD28-	19.9 (8.2- 24.3)	12.2 (6.6- 22.1)	10.7 (4.8- 24.1)	0.5	0.4	0.7
CD4+ CD27-/CD28+	16.1 (8.8- 17.6)	10.0 (8.9- 14.5)	8.1 (7.5- 12.9)	0.14	0.089	0.2
CD4+ CD27+/CD28-	3.6 (2.6- 9.5)	5.1 (2.2- 10.4)	5.9 (1.4- 13.9)	>0.9	>0.9	0.9
CD4+ CD27+/CD28+	55.6 (44.0- 69.7)	72.3 (40.6- 78.9)	74.0 (54.3- 79.0)	0.7	0.4	0.7
CD4+ PD-1-/CD57-	41.9 (35.4- 50.2)	51.3 (48.7- 65.8)	58.9 (42.6- 64.9)	**0.046**	0.068	>0.9
CD4+ PD-1-/CD57+	4.7 (1.1- 8.8)	5.0 (1.5- 12.2)	5.3 (1.0- 8.7)	0.7	>0.9	0.7
CD4+ PD-1+/CD57-	39.3 (32.3- 46.6)	29.9 (17.0- 40.4)	25.7 (15.7- 39.1)	0.087	0.10	0.6
CD4+ PD-1+/CD57+	9.1 (3.2- 15.0)	4.2 (2.9- 9.6)	6.4 (3.0- 11.1)	0.3	0.7	0.5
Treg (CD25+/CD127 low) event count	357.5 (184.5- 397.5)	330.0 (293.0- 465.0)	461.5 (369.0- 563.5)	0.6	0.14	0.2
Treg (CD25+/CD127 low)	23.5 (9.1- 34.3)	13.0 (10.4- 18.5)	14.1 (12.1- 17.4)	0.3	0.4	0.8
Treg (CD25low/CD127low)	10.6 (7.5- 14.9)	14.3 (11.4- 21.7)	13.7 (13.1- 21.1)	0.10	0.2	>0.9
Th 1-2 DN	15.1 (11.4- 20.0)	11.7 (9.6- 26.7)	11.7 (8.9- 18.6)	0.6	0.4	0.9
Th1	21.5 (17.7- 34.5)	19.3 (18.4- 26.3)	22.3 (16.0- 29.8)	>0.9	>0.9	>0.9
Th1-2	2.9 (2.0- 4.1)	5.2 (3.3- 7.4)	5.5 (2.9- 8.7)	0.2	0.2	0.7
Th2	2.7 (1.8- 4.0)	3.3 (2.9- 4.6)	3.6 (2.3- 5.0)	0.4	0.4	0.8
Th17 (CCR4+)	6.0 (3.8- 8.5)	7.4 (5.9- 10.0)	8.2 (6.0- 10.3)	0.3	0.2	>0.9
Th17-1 (CXCL3+ CCR4+)	11.7 (7.5- 19.6)	10.4 (6.7- 20.1)	11.0 (7.4- 18.7)	>0.9	0.9	0.7
Th17.1 (CXCL3+)	16.5 (10.4- 19.0)	17.0 (10.2- 23.8)	15.3 (12.9- 17.8)	0.8	>0.9	0.8
Th17DN	3.6 (2.7- 5.9)	4.0 (2.8- 5.5)	3.5 (2.6- 4.4)	0.8	0.6	0.3
CD8 event count	4,688.5 (3,079.5- 5,587.8)	4,575.0 (4,181.0- 5,794.0)	4,233.0 (3,777.5- 5,365.5)	0.5	>0.9	0.5
% CD8+ on CD3	53.6 (33.1- 57.3)	50.1 (45.5- 58.2)	44.7 (41.8- 54.9)	0.5	0.8	0.3
CD8+ EM1	17.1 (11.3- 31.4)	14.1 (7.6- 19.4)	11.8 (7.5- 13.5)	0.3	0.13	0.5
CD8+ EM2	10.0 (8.8- 19.4)	12.7 (12.1- 20.8)	11.7 (10.3- 15.9)	0.3	0.6	0.5
CD8+ EM3	21.9 (9.3- 29.1)	18.7 (7.8- 25.6)	19.9 (8.1- 26.7)	0.8	>0.9	0.9
CD8+ EM4	1.9 (1.2- 2.2)	1.4 (0.9- 2.3)	1.4 (1.2- 2.1)	0.7	0.8	>0.9
CM-CD8+	8.2 (6.9- 10.0)	4.2 (3.3- 5.7)	4.2 (2.5- 6.5)	**0.007**	**0.020**	0.9
EM-CD8+	56.7 (49.7- 61.7)	50.0 (45.5- 57.4)	45.7 (38.5- 53.8)	0.3	0.11	0.3
N-CD8+	6.3 (3.7- 9.2)	6.5 (4.5- 9.6)	7.4 (6.1- 10.0)	>0.9	0.4	0.5
TEMRA-CD8+	29.9 (21.9- 34.8)	38.7 (24.3- 41.7)	39.6 (30.0- 46.4)	**0.035**	**0.045**	0.6
CD8+ CD27-/CD28-	37.7 (29.9- 48.9)	43.7 (36.6- 52.8)	47.9 (37.8- 54.7)	0.2	0.2	0.9
CD8+ CD27-/CD28+	2.2 (1.6- 3.4)	2.6 (1.1- 2.8)	2.2 (1.9- 3.0)	0.9	>0.9	>0.9
CD8+ CD27+/CD28-	20.8 (11.5- 23.5)	19.8 (16.1- 24.8)	19.5 (13.9- 27.1)	0.8	0.7	>0.9
CD8+ CD27+/CD28+	34.9 (27.4- 50.5)	28.8 (19.6- 47.3)	27.8 (15.8- 35.9)	0.4	0.2	0.8
CD8+ PD-1-/CD57-	29.3 (23.2- 34.4)	40.2 (31.9- 48.2)	38.5 (27.3- 47.3)	**0.014**	0.14	0.7
CD8+ PD-1-/CD57+	29.5 (14.5- 36.8)	29.4 (24.2- 35.9)	30.3 (23.7- 44.3)	0.5	0.4	0.7
CD8+ PD-1+/CD57-	33.5 (22.3- 40.9)	22.9 (11.5- 25.5)	18.7 (11.0- 24.9)	**0.046**	**0.020**	0.5

CD8+ PD-1+/CD57+	9.9 (5.6- 12.2)	4.8 (3.9- 8.4)	7.3 (4.1- 9.5)	0.060	0.3	0.6
RATIO CD4/CD8	0.7 (0.6- 1.1)	0.7 (0.6- 1.0)	0.8 (0.7- 1.0)	>0.9	0.7	0.6
IL-4 pg/ml	43 [39- 76.2]	44 [39- 90.6]	39.6 [39.18- 43.5]	>0.9	0.2	0.3
IL-6 pg/ml	108.8 [6-798]	22.35 [6-162]	43.53 [6-272]	0.2	0.3	0.9

Continuous variables are expressed as median of percentage frequency % (IQR). Cytokine levels were represented as mean and [min-max]. [1]Wilcoxon signed rank test for paired data with false discovery rate correction (fdr).

Table S2: B and T cell subtype frequencies according to the HIV-coinfection and the time from SO

	T0			T1			T2			
**Characteristics**	**HIV-** N = 9	**HIV+** N = 3	**p-value/**	**HIV-** N = 9	**HIV+,** N = 4	**p-value/**	**HIV-** N = 8	**HIV+** N = 4	**p-value/**	**HD**
B event count	1,000.0 (552.0-1,025.0)	1,017.0 (810.0-1,031.0)	0.6	1,049.0 (1,016.0-1,094.0)	899.5 (779.5-1,275.5)	0.3	1,033.5 (1,020.2-1,053.0)	1,334.0 (987.5-1,713.2)	0.7	1,061 (1,035-1,249)
% B cells on Leuko	0.9 (0.7- 1.5)	2.6 (1.8- 3.0)	0.1	2.5 (1.9- 3.2)	1.9 (1.3- 2.8)	0.7	2.3 (1.7- 3.3)	2.1 (1.4- 3.0)	0.4	2.49 (1.77-4.20)
Active MB (IgD-/CD27+/CD21-)	18.4 (12.9- 27.6)	43.0 (37.9- 65.6)	**0.018**	18.5 (15.4- 19.7)	32.1 (25.3- 37.3)	0.2	16.2 (13.4- 19.3)	20.1 (14.4- 32.2)	0.7	4.89 (3.5-14.78)
B reg (CD24 high/CD38 high)	2.2 (1.1- 3.3)	4.1 (3.7- 5.9)	0.15	1.4 (0.7- 3.1)	0.7 (0.5- 2.0)	0.9	0.8 (0.4- 1.5)	1.4 (1.1- 1.7)	0.4	2.61 (1.78-3.71)
CD27-	47.6 (37.3- 58.2)	43.3 (26.0- 44.9)	0.3	45.9 (40.1- 59.6)	51.5 (43.4- 59.8)	0.4	47.2 (39.7- 51.0)	65.4 (47.9- 75.5)	0.2	55.07 (44.96-63.24)
CD27+	52.4 (41.5- 62.7)	56.9 (55.2- 74.1)	0.9	53.9 (40.4- 60.1)	48.8 (40.3- 56.9)	0.4	52.8 (49.0- 60.5)	34.4 (24.1- 52.1)	0.2	44.37 (35.54-54.60)
DN (IgD-/CD27-)	8.7 (7.0- 11.6)	7.6 (4.9- 7.6)	0.6	8.1 (5.6- 8.5)	8.6 (7.7- 9.7)	0.4	7.6 (5.5- 8.8)	8.6 (7.9- 10.3)	0.4	1.41 (0.12-2.93)
MZ (CD27+/IgD^{+})	17.2 (13.4- 27.4)	44.2 (38.2- 67.1)	**0.018**	18.7 (17.1- 22.2)	29.5 (24.6- 31.6)	0.2	19.8 (17.1- 24.3)	17.9 (14.1- 30.0)	0.8	42.01 (20.83-45.15)
Memory B (IgD-/CD27+)	32.3 (10.5- 38.0)	6.1 (4.1- 17.4)	>0.9	36.6 (19.2- 40.2)	19.8 (12.7- 31.1)	0.3	35.2 (28.0- 41.3)	18.3 (13.3- 23.8)	**0.048**	9.45 (0.13-16.07)
Naive (IgD+/CD27-)	46.0 (30.5- 58.0)	31.6 (18.5- 36.8)	0.2	40.2 (35.3- 49.6)	38.8 (31.8- 47.5)	0.6	36.2 (29.3- 42.0)	50.4 (36.5- 59.3)	0.4	54.88 (40.04-63.94)
plasmablasts (SWI/CD38+)	0.7 (0.4- 2.5)	0.3 (0.1- 0.7)	0.7	0.5 (0.3- 2.9)	0.2 (0.1- 0.3)	0.3	0.2 (0.2- 0.7)	0.8 (0.7- 1.0)	0.4	0.1 (0.0-0.24)
Resting MB (IgD-/CD27+/CD21+)	15.6 (13.2- 28.1)	6.8 (4.8- 12.3)	0.15	31.4 (22.7- 35.9)	12.4 (7.2- 19.3)	**0.031**	31.3 (24.5- 40.5)	12.0 (7.9- 16.3)	**0.016**	37.30 (15.51-45.84)
SWIB (CD27+/ IgD-/IgM-)	19.3 (17.0- 31.5)	11.2 (7.7- 20.7)	0.3	35.6 (19.8- 41.1)	26.1 (20.0- 35.5)	0.5	37.6 (30.4- 46.2)	25.4 (22.1- 27.5)	0.2	14.22 (4.13-28.14)
TLM (CD21-/CD27-)	13.3 (9.1- 21.6)	10.6 (7.5- 15.7)	0.7	8.6 (7.1- 8.8)	15.4 (14.3- 18.1)	**0.003**	8.5 (7.5- 9.1)	15.7 (10.3- 25.8)	**0.028**	6.18 (3.86-12.02)
Trans B (CD27-/CD24+/CD38+)	1.8 (0.5- 3.8)	1.8 (1.4- 2.9)	0.4	2.2 (0.3- 2.5)	0.2 (0.1- 1.1)	0.6	0.5 (0.1- 1.1)	0.9 (0.3- 1.5)	0.7	1.95 (1.17-2.50)
UNSW (CD27+/IgD+/IgM+)	62.2 (52.9- 75.4)	78.3 (67.8- 86.5)	0.3	60.7 (54.7- 74.8)	55.0 (47.2- 63.3)	0.6	58.6 (50.0- 65.6)	65.8 (63.8- 70.0)	0.2	73.39 (40.04-83.29)
CD4 event count	3,416 (2,947-3,706)	3,227 (2,222, 3,330)	0,6	3,542 (2,824, 3,780)	3,426 (2,968, 3,704)	0,7	3,654 (3,263, 4,607)	3,858 (3,028, 4,322)	0,8	5,984 (5,236-6,331)
% CD4 on CD3	36.8 (31.9- 45.3)	31.9 (25.0- 32.1)	0.2	43.4 (35.4- 46.5)	36.0 (31.2- 39.0)	0.3	38.5 (35.2- 45.6)	40.4 (35.4- 41.3)	0.8	59.5 (52.8- 61.4)
CD4+ EM1	24.4 (17.0- 30.4)	16.4 (16.0- 19.6)	0.4	22.9 (22.0- 31.6)	34.9 (24.4- 44.9)	0.3	25.3 (18.7- 28.2)	46.8 (36.0- 50.6)	0.2	20.9 (19.8- 24.8)
CD4+ EM2	3.1 (1.1- 10.7)	1.4 (0.7- 3.3)	0.4	4.9 (1.1- 12.9)	0.7 (0.5- 1.3)	**0.034**	6.1 (1.9- 11.9)	1.8 (0.9- 4.4)	0.5	0.2 (0.1- 0.3)
CD4+ EM3	11.4 (3.8- 16.4)	16.7 (8.8- 22.5)	0.7	12.0 (6.9- 14.4)	4.2 (1.9- 12.7)	0.3	10.9 (4.1- 18.5)	4.3 (2.9- 13.1)	0.8	3.8 (0.0- 7.1)
CD4+ EM4	7.5 (6.3- 10.5)	11.9 (8.2- 13.9)	0.6	7.5 (6.4- 10.7)	12.6 (9.6- 14.5)	0.3	7.4 (5.2- 9.2)	9.7 (7.0- 12.5)	0.4	7.4 (6.2- 9.0)
CM-CD4+	24.2 (20.6- 35.1)	42.2 (27.2- 44.2)	0.5	20.9 (16.7- 34.9)	18.1 (11.7- 25.5)	0.5	28.7 (15.0- 41.1)	10.5 (9.5- 12.4)	0.2	43.6 (39.9- 48.6)
EM-CD4+	57.7 (40.5- 62.6)	45.5 (37.0- 55.7)	0.7	52.5 (41.4- 68.1)	63.6 (51.4- 69.5)	>0.9	50.4 (35.6- 62.4)	64.5 (61.2- 68.0)	0.2	33.8 (31.5- 38.5)
N-CD4+	8.3 (5.9- 19.7)	9.9 (8.0- 17.0)	0.7	11.4 (7.6- 15.0)	13.5 (9.6- 19.4)	0.6	12.8 (9.8- 14.8)	11.9 (11.0- 13.1)	>0.9	18.4 (13.8- 23.8)
TEMRA-CD4+	6.1 (2.1- 10.9)	2.4 (1.9- 9.0)	>0.9	6.6 (3.4- 11.8)	5.4 (4.4- 9.0)	>0.9	5.4 (2.2- 7.4)	11.4 (7.2- 15.8)	0.11	2.0 (0.8- 2.8)
CD4+ CD27-/CD28-	19.3 (7.8- 23.9)	20.5 (14.5- 33.6)	0.6	16.9 (10.3- 22.1)	6.7 (5.9- 16.0)	0.6	15.1 (7.4- 24.1)	4.7 (3.2- 15.1)	0.5	4.2 (0.0- 7.1)
CD4+ CD27-/CD28+	16.0 (7.7- 16.3)	18.8 (14.0- 20.8)	0.4	9.1 (8.3- 10.5)	13.8 (11.2- 16.3)	0.11	8.1 (6.9- 10.2)	10.9 (7.9- 13.8)	0.6	10.7 (7.3- 12.1)
CD4+ CD27+/CD28-	3.9 (3.1- 13.3)	1.8 (1.6- 3.5)	0.3	6.4 (3.7- 10.7)	2.0 (1.6- 3.1)	0.076	8.6 (3.0- 15.9)	2.2 (0.9- 6.0)	0.2	0.3 (0.2- 0.4)
CD4+ CD27+/CD28+	56.2 (44.8- 65.9)	54.9 (42.1- 67.9)	0.6	61.0 (40.6- 76.7)	79.5 (65.9- 82.1)	0.3	64.0 (54.3- 77.3)	79.2 (67.6- 82.5)	0.3	84.0 (79.3- 92.3)
CD4+ PD-1-/CD57-	43.1 (40.7- 50.7)	29.7 (28.8- 32.2)	**0.009**	57.8 (48.7- 66.0)	50.1 (47.1- 54.5)	0.4	61.2 (53.5- 64.9)	44.0 (34.4- 57.6)	0.3	70.1 (58.3- 78.9)
CD4+ PD-1-/CD57+	6.6 (1.1- 8.4)	1.5 (0.9- 9.0)	>0.9	5.8 (1.6- 12.2)	1.7 (1.3- 9.3)	0.7	6.5 (2.8- 8.7)	2.6 (0.3- 11.9)	0.5	1.3 (0.3- 2.3)
CD4+ PD-1+/CD57-	36.1 (33.9- 43.3)	54.1 (40.8- 57.7)	0.3	29.9 (14.8- 40.4)	33.7 (26.1- 40.4)	0.6	25.7 (22.2- 33.4)	34.5 (8.2- 61.6)	>0.9	24.8 (19.3- 39.0)
CD4+ PD-1+/CD57+	7.7 (1.9- 10.8)	14.8 (9.3- 21.4)	0.4	4.2 (2.8- 7.3)	6.5 (3.3- 10.1)	0.6	5.1 (3.0- 12.8)	7.4 (6.0- 8.5)	>0.9	2.5 (0.9- 7.0)

Treg (CD25+/CD127 low) event count	276.0 (177.0- 369.0)	904.0 (656.0- 1-131.5)	**0.009**	323.0 (198.0- 397.0)	483.5 (408.8- 565.2)	0.15	461.5 (308.5- 524.5)	484.5 (396.8- 583.5)	0.6	50.0 (25.0-112.0)
Treg (CD25+/CD127 low)	11.0 (6.9- 33.3)	30.3 (28.4- 33.8)	0.04	11.6 (9.2- 17.3)	17.7 (13.4- 22.6)	0.15	12.8 (10.1- 16.3)	16.8 (15.8- 17.4)	0.4	3.4 (2.6- 4.6)
Treg (CD25low/CD127low)	8.4 (6.9- 14.8)	13.3 (10.5- 18.1)	0.5	14.3 (12.3- 21.7)	13.0 (9.7- 20.9)	0.7	13.5 (11.7- 15.9)	20.3 (13.6- 28.2)	0.2	11.0 (8.2- 17.6)
Th 1-2 DN	15.6 (11.8- 23.2)	14.5 (12.3- 15.7)	0.7	11.7 (9.9- 26.7)	10.3 (4.3- 24.2)	0.7	10.6 (8.9- 14.0)	17.7 (13.5- 24.8)	0.6	11.1 (7.4- 13.7)
Th1	22.5 (17.8- 34.4)	17.9 (15.1- 36.6)	>0.9	24.4 (19.3- 26.3)	16.7 (14.6- 18.5)	**0.011**	24.8 (19.5- 39.4)	19.3 (15.0- 22.5)	0.4	14.6 (12.6- 18.7)
Th1-2	3.4 (2.3- 4.0)	2.0 (1.7- 6.0)	0.6	5.4 (3.7- 7.4)	2.9 (2.3- 4.7)	0.3	5.8 (4.0- 8.7)	4.4 (2.1- 7.5)	0.6	5.7 (4.3- 7.6)
Th2	2.7 (1.9- 4.8)	2.2 (1.8- 3.0)	0.6	4.4 (3.0- 5.0)	3.1 (2.7- 3.3)	0.3	3.6 (2.1- 4.6)	4.0 (3.0- 5.2)	0.8	5.1 (4.0- 6.7)
Th17 (CCR4+)	6.1 (4.2- 9.8)	6.0 (4.0- 6.8)	0.6	7.4 (6.5- 10.0)	7.6 (5.8- 9.5)	0.6	7.1 (6.0- 9.3)	9.7 (8.3- 10.3)	0.7	13.8 (10.9- 15.3)
Th17-1 (CXCL3+ CCR4+)	10.5 (7.8- 14.1)	22.2 (13.4- 22.2)	0.6	8.3 (6.7- 13.6)	23.0 (17.5- 26.0)	0.2	11.5 (7.4- 18.7)	11.0 (9.0- 18.0)	>0.9	13.9 (11.5- 16.0)
Th17.1 (CXCL3+)	17.6 (12.1- 19.0)	9.6 (9.0- 16.8)	0.7	17.0 (11.2- 23.8)	16.4 (9.4- 24.7)	>0.9	15.8 (14.9- 18.6)	11.1 (8.0- 15.6)	0.2	18.0 (16.0- 23.3)
Th17DN	3.7 (3.1- 5.7)	2.2 (1.7- 6.0)	0.6	4.0 (2.8- 5.5)	4.6 (3.6- 5.7)	0.8	3.2 (2.5- 4.1)	4.3 (3.3- 5.3)	0.4	7.6 (5.5- 9.7)
CD8 event count	4,015 (1,092- 5,460)	5,635 (4,990- 5,720)	0.2	4,575 (3,755- 5,794)	4,796 (4,553- 5,303)	0.7	4,233 (3,881- 5,558)	4,394 (3,778- 5,056)	0.8	3,130 (2,814-3,729)
% CD8 on CD3	44.4 (20.6- 55.4)	57.3 (55.1- 61.2)	0.2	48.7 (44.8- 57.9)	54.2 (49.9- 59.0)	0.4	44.1 (41.0- 55.5)	47.9 (43.2- 53.0)	0.8	31.1 (28.8- 35.5)
CD8+ EM1	16.2 (9.8- 31.0)	19.6 (17.2- 26.5)	0.5	9.6 (7.0- 18.8)	22.6 (13.8- 31.1)	0.15	11.3 (7.1- 12.3)	14.4 (11.2- 20.3)	0.2	22.0 (15.2- 29.4)
CD8+ EM2	10.2 (8.7- 18.9)	9.8 (9.3- 15.2)	>0.9	12.7 (12.1- 25.1)	10.5 (7.6- 13.5)	0.3	13.5 (11.8- 22.9)	9.6 (7.2- 11.1)	**0.028**	5.4 (3.9- 6.5)
CD8+ EM3	23.5 (10.6- 27.8)	14.2 (9.4- 24.3)	>0.9	19.5 (17.7- 26.6)	11.9 (5.1- 19.2)	0.2	24.5 (15.9- 28.6)	9.7 (5.5- 15.7)	0.073	11.6 (3.0- 29.9)
CD8+ EM4	1.2 (1.1- 2.3)	2.0 (1.9- 2.1)	0.6	1.2 (0.5- 1.8)	2.1 (1.6- 2.7)	0.3	1.4 (1.1- 1.8)	1.7 (1.3- 2.1)	0.6	3.8 (2.3- 5.3)
CM-CD8+	9.6 (7.3- 10.0)	7.5 (4.7- 7.6)	0.2	4.1 (3.3- 4.7)	4.9 (3.7- 6.7)	0.6	5.1 (3.2- 7.4)	2.6 (2.2- 3.6)	0.3	6.7 (4.9- 10.7)
EM-CD8+	57.0 (50.1- 63.0)	56.5 (52.5- 58.9)	0.9	50.8 (48.2- 57.4)	46.5 (42.6- 51.4)	0.3	50.8 (42.6- 60.1)	39.2 (35.5- 42.7)	0.11	56.2 (40.0- 59.7)
N-CD8+	6.8 (5.2- 15.6)	3.8 (3.6- 5.4)	0.4	6.5 (5.4- 9.9)	6.5 (3.9- 9.0)	0.5	7.4 (5.7- 8.9)	8.1 (6.6- 10.2)	0.8	20.0 (14.8- 30.5)
TEMRA-CD8+	29.3 (19.4- 30.5)	36.9 (32.1- 37.5)	0.3	38.6 (23.6- 39.9)	43.2 (36.2- 47.7)	0.2	31.0 (28.5- 37.7)	47.5 (43.9- 52.7)	0.073	17.6 (5.8- 25.3)
CD8+ CD27-/CD28-	36.5 (30.9- 47.7)	38.9 (33.0- 47.2)	0.9	49.3 (42.0- 57.4)	40.2 (34.8- 46.0)	0.6	43.8 (37.5- 55.5)	50.2 (45.3- 52.5)	0.8	20.4 (4.0- 49.2)
CD8+ CD27-/CD28+	2.0 (1.5- 3.5)	2.4 (2.3- 2.9)	0.5	1.9 (0.9- 2.8)	3.2 (2.4- 4.0)	0.3	2.1 (1.4- 2.9)	2.4 (2.1- 3.5)	0.4	4.2 (2.4- 7.0)
CD8+ CD27+/CD28-	20.9 (9.4- 27.5)	20.7 (16.5- 21.4)	>0.9	20.2 (16.1- 24.8)	16.9 (15.0- 19.7)	0.6	21.5 (13.9- 37.2)	17.7 (14.9- 20.4)	0.6	7.1 (4.9- 8.6)
CD8+ CD27+/CD28+	33.1 (23.2- 52.6)	36.6 (32.7- 43.2)	>0.9	21.4 (14.2- 37.9)	38.0 (28.4- 48.1)	0.4	21.7 (14.7- 32.3)	32.7 (28.5- 35.9)	0.4	62.6 (38.2- 78.5)
CD8+ PD-1-/CD57-	28.9 (21.1- 34.2)	30.8 (27.4- 32.9)	0.7	40.2 (31.9- 50.8)	39.6 (36.2- 43.3)	>0.9	38.5 (27.1- 51.5)	37.5 (31.0- 42.6)	>0.9	51.3 (33.4- 70.9)
CD8+ PD-1-/CD57+	26.9 (14.1- 36.0)	33.8 (24.2- 42.0)	0.5	29.4 (24.2- 32.7)	33.4 (26.1- 38.3)	0.8	27.0 (23.7- 35.7)	38.0 (29.2- 44.3)	0.7	12.2 (7.0- 31.5)
CD8+ PD-1+/CD57-	33.8 (23.4- 43.4)	23.9 (18.9- 32.0)	0.6	22.9 (11.5- 25.5)	22.0 (17.4- 26.1)	>0.9	18.7 (14.4- 22.8)	17.6 (10.1- 26.1)	>0.9	20.3 (16.0- 28.8)
CD8+ PD-1+/CD57+	7.9 (5.4- 12.7)	11.5 (10.9- 11.7)	0.5	4.7 (3.0- 5.9)	8.7 (7.2- 9.2)	0.2	5.8 (3.8- 9.0)	8.3 (6.6- 11.8)	0.6	4.8 (3.4- 8.6)
RATIO CD4/CD8	0.8 (0.7- 1.6)	0.6 (0.4- 0.6)	0.1	0.9 (0.6- 1.1)	0.7 (0.5- 0.8)	0.3	0.8 (0.7- 1.1)	0.9 (0.7- 1.0)	0.8	1.9 (1.4-2.0)

Categorical variables are expressed as n/N. Continuous variables are expressed as the median of relative frequency % (IQR). [1] Wilcoxon Mann-Whitney test. Significant p-values are shown in bold.
