# Supplementary material for: Early Diagnosis and Monitoring of Adaptive Immune Response in a Cohort of Mild Mpox Patients During the 2022 Wave
Source: Microorganisms. 2025 Feb 6;13(2):355. doi: 10.3390/microorganisms13020355 (PMC11858686; doi:10.3390/microorganisms13020355)
Supplement: Supplementary file 1 [file microorganisms-13-00355-s001.zip › Caldrer_Supplementary Figures_v.3.pdf]

Supplementary figures to the manuscript entitled: Early diagnosis and monitoring of adaptive immune response in a cohort of mild mpox patients during the 2022 wave

By Sara Caldrer[1*], Silvia Accordini[1], Annalisa Donini[1], Gianesini Natasha[1], Andrea Matucci[1], Antonio Mori[1], Cristina Mazzi[2], Maddalena Cordioli[3,4], Evelina Tacconelli[4], Niccolò Ronzoni[1], Andrea Angheben[1], Chiara Piubelli[1], Gobbi Federico[1], Concetta Castilletti[1*]

Supplementary Figures

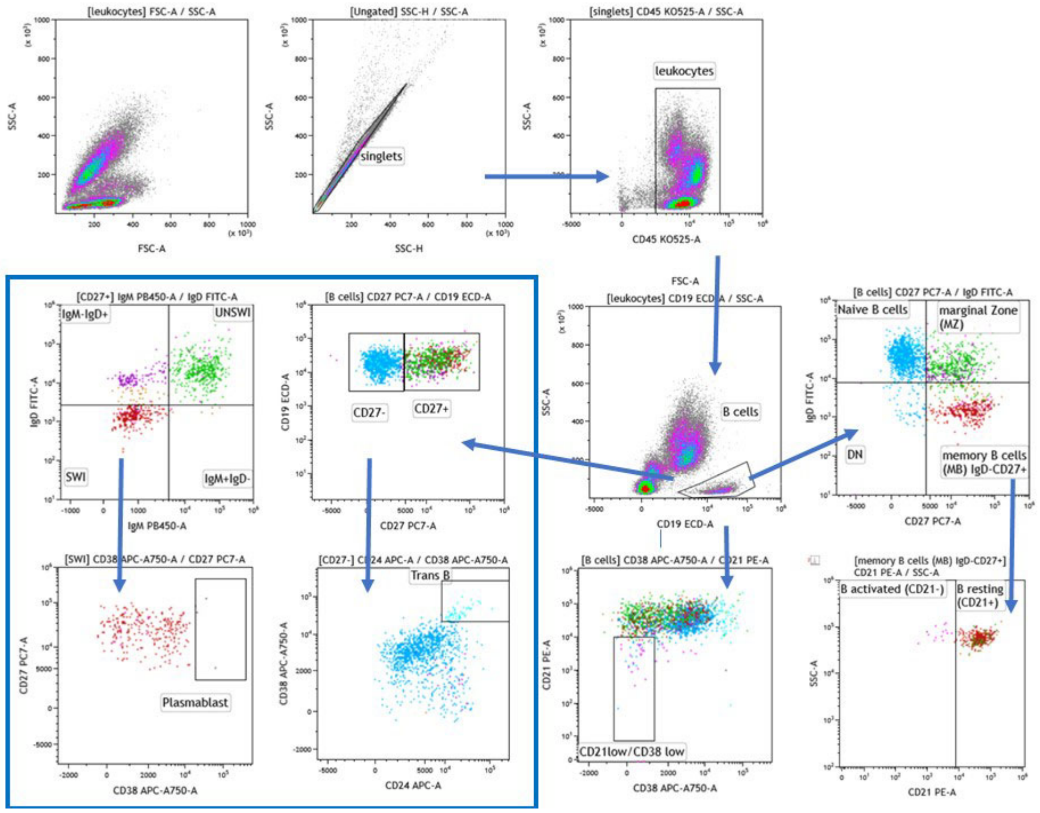

Supplementary Fig. S1. Gating strategy for B cell phenotypes. Dot plots used for the gating strategy for B-cell subsets. Aggregate exclusion was performed by single cell selection and Krome Orange KO525 gating for CD45+ leukocytes. B-cells were then selected by CD19-ECD expression. Therefore, different gating strategies were employed to identify the different B subpopulations.

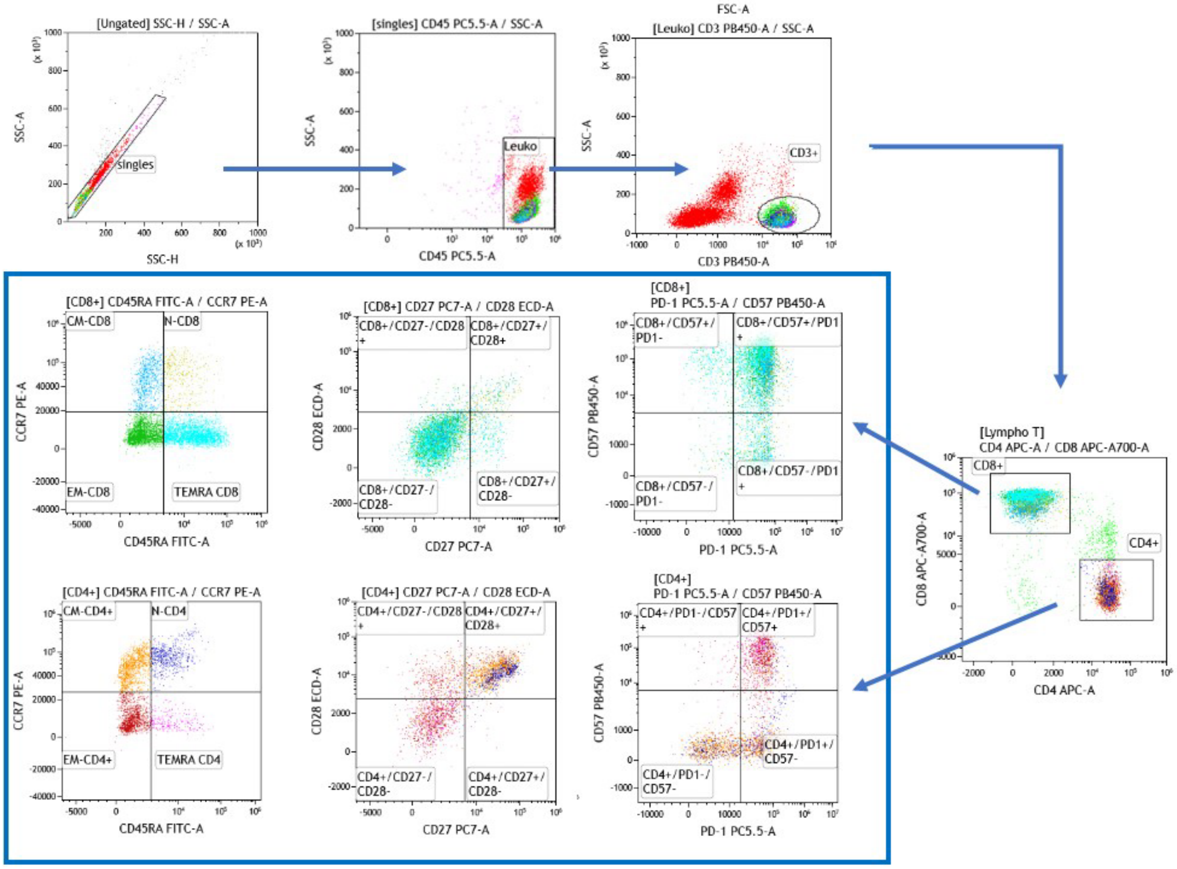

Supplementary Fig. S2. Gating strategy for CD4 and CD8 T-cells phenotypes. Gating strategy for T-cell subsets. Aggregate exclusion was achieved through single-cell selection, forward-scatter (FSC) versus side-scatter (SSC) gating, and Krome Orange KO525 gating for CD45+ cells (leukocytes). Lymphocytes (T-cells) were then identified by CD3-APC750 expression. Additionally, CD8-AF700 and CD4-APC were employed to differentiate T cytotoxic cells and T helper lymphocytes, respectively. Consequently, different presentation and gating were used to discern other different T-cell subpopulations.

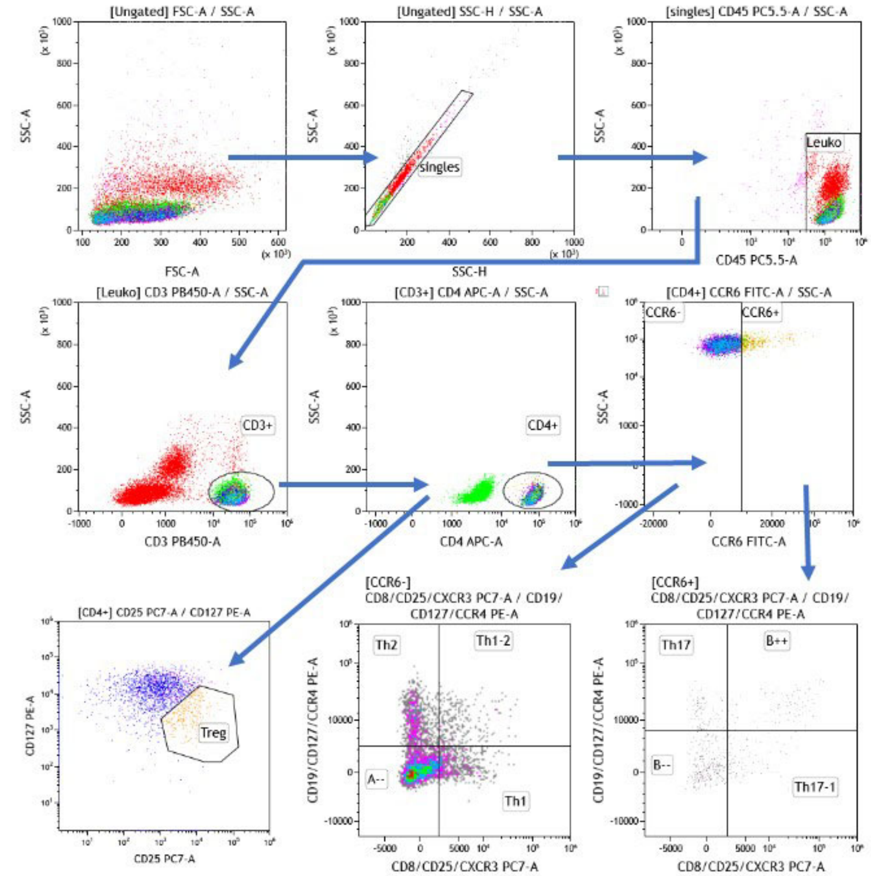

Supplementary Fig. S3. Gating strategy for regulatory and helper T-cells phenotypes. Gating strategy adopted for the enumeration of selected cell targets by multi-colour flow cytometry. Lymphocytes, monocytes and granulocytes were determined based on the FSC on the CD45^{+} leucocyte gate. T lymphocyte subsets were identified as CD3^{+} and then differentiated in CD4^{+} or CD8^{+}. Finally, CD4^{+} T-cell subsets were identified as follows: Th1 (CD4^{+}CCR6^{-}CXCR3^{+}); Th2 (CD4^{+}CCR6^{-}CCR4^{+}); Th17 (CD4^{+}CCR6^{+}CCR4^{+}); Th17-1 (CD4^{+}CCR6^{+}CXCR3^{+}); Treg (CD4^{+}CD25^{+}CD127^{low}).

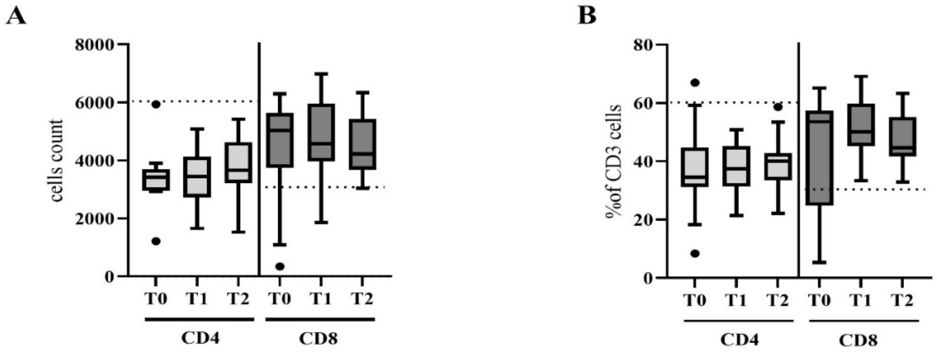

Supplementary Fig. S4. CD4+ and CD8+ T cells count and frequency during MPXV infection. Barr plot represents the median and IQR values of each T-cell subpopulation in relation to the time point from SO. The dashed line represents the median value obtained from HD. Statistical differences among groups were determined using the Wilcoxon rank sum test.
